# Aortic Valve Repair as a Subspecialty: Still an Institutional Practice or Open for All?

**DOI:** 10.21470/1678-9741-2021-0160

**Published:** 2022

**Authors:** Kaushalendra Rathore

**Affiliations:** 1 Department of Cardiothoracic Surgery, Sir Charles Gairdner Hospital, Nedlands, Australia.

**Keywords:** Aortic Valve, Heart Valve Diseases, thnicity, Learning Curve, Communication, Referral and Consultation

## Abstract

Aortic valve repair combined with root stabilization procedures have been reported to have reliable mid to long-term outcomes, and this is one of the reasons that various surgical units are accepting these techniques as an option in selected cases. Aortic valve replacement is a standard procedure with established results, but to master its techniques there is a major uphill learning curve. A brief communication is presented on the aortic valve repair focusing on the lesser discussed aspects like global variability of the pathology and outcomes, variable referral patterns, and effect of ethnicity.

**Table:** Abbreviations, Acronyms & Symbols

AR	= Aortic regurgitation
AS	= Aortic stenosis
AV	= Aortic valve
AVIATOR	= Aortic Valve Insufficiency and ascending aorta Aneurysm InternATiOnal Registry
AVr	= Aortic valve repair
AVR	= Aortic valve replacement
BAV	= Bicuspid aortic valve
CTD	= Connective tissue disease
FAA	= Functional aortic annulus
IE	= Infective endocarditis
RHD	= Rheumatic heart disease
SOV	= Sinus of Valsalva
STJ	= Sinotubular junction
SVD	= Structural valve deterioration
TOE	= Transoesophageal echocardiogram
VSD	= Ventricular septal defect

## INTRODUCTION

Aortic valvular disease is a global public health hazard, and aortic pathologies have significant presence in all demographic groups, with overall increase in the ageing population^[1]^. Severely diseased aortic valves (AVs) are best managed with surgical interventions, and available surgical options are replacement using mechanical or bioprosthetic valve, homograft, Ross procedure using pulmonary autograft, or aortic valve repair (AVr). Aortic valve replacement (AVR) is a well-established procedure with proven long-term outcomes, but patients are constantly exposed to the risk of anticoagulation-related bleeding, valve thrombosis, structural valve deterioration (SVD), pregnancy-related issues, and infections following prosthetic implantation^[2]^. Freedom from valve-related reoperation favoured mechanical valves for all age groups for fifteen years, except for patients aged > 60 years^[3]^. Similarly, fifteen-year freedom from the valve-related morbidity favoured biologic valves for all age groups, except in patients < 40 years of age at operation.

Interpretation of large contemporary literature reveals that bioprosthetic valves appeared to be favourable in patients on the basis of the lower incidence of valve-related morbidity, but they are associated with increased overall valve-related reoperations (the actual freedom from valve-related reoperation in patients between 51-60 years were 98.3% and 59.7% for the mechanical and bioprosthetic groups, respectively)^[4]^. Une et al.^[5]^ have reported good durability of the Hancock II aortic bioprosthesis up to the 10-year follow-up following the implantation in young patients (91.4% actuarial freedom from re-AVR due to SVD)^[5]^. Their series showed that SVD significantly increased from 10 years to 20 years after surgery, especially in patients aged < 50 years and, at the 20-year follow-up, actuarial freedom from re-AVR due to SVD was 41.4% in patients aged 50-59 years.

To reduce disadvantages of the bioprosthesis in the younger population, surgical community progressed towards the repairing options. It was surely influenced by the success of the mitral valve repair that AV, ascending aorta, and aortic arch became the next field of interest in last couple of decades. Pioneers like Yacoub, Davids, and El Khoury et al. have described various repair techniques with good long-term outcomes, which completely revolutionized aortic surgery and helped in developing it as a subspecialty^[6-8]^. With all supporting contemporary literature, a larger question still remains about its applicability, acceptance, and generalization of the indications, as the reoperation rate, even in high-volume centres, following AVr is approximately 10.23% within four years of follow-up^[8]^.

## COMMENTS

### What is Driving AVr?

It is well stated that AVR with bioprostheses in young adults is associated with high rates of SVD and reintervention; in patients aged between 20 to 40 years, one or more reinterventions during their lifetime are anticipated^[9]^. Although early mortality is low, the long-term survival is reduced, with a life expectancy of 60% to 75% of the age- and sex-matched life expectancy of general population. Etnel et al.^[9]^ have reported that bioprosthetic AVR in young adults is associated with low early mortality (3.30%), but the late mortality is high (2.39%/year), and thus overall life expectancy is impaired compared with the general population. Their study showed that thromboembolism (0.53%/year) and bleeding (0.22%/year) rates are far lower than reported for mechanical AVR in young adults (0.90%/year and 0.85%/year, respectively). Others have also reported that after AVR, thromboembolism and bleeding rates were higher compared to the general population and higher than after the Ross procedure and AVr^[10]^. On the other side, AVr is consistently associated with a low risk of late valve-related morbidity, thromboembolism (< 0.7%/patient year), bleeding (< 0.3%/patient year), and infective endocarditis (< 0.2%/patient year)^[7,11]^. Overall freedom from reoperation was 95% for tricuspid valves at 10 and 15 years, 89% for bicuspid aortic valves (BAV) at 10 years, and 83% for BAV at 15 years. These results are encouraging for a more widespread use of AVr, but undeniable factors like case selection and institutional case volume make a significant difference in the long-term outcomes.

### Why is AVr Challenging?

AV is considered one of the parts of several components of the aortic root functioning unit and to achieve good repair outcomes, the surgeon has to address lesions at multiple levels, *e.g.*, sinotubular junction (STJ), functional aortic annulus (FAA), sinus of Valsalva (SOV), and AV simultaneously (Table 1). In the initial experience, only FAA was in focus, and leaflet issues were remained unaddressed, but as experience grew, surgical groups identified the importance of leaflets, SOV, and the role of valvular pathology, respectively^[12]^. Basic concept of good repair is to increase free margin length with maximising coaptation height, and performing all these additional procedures not only increases myocardial ischemic time but also increases chances of repair failure^[13]^.

**Table 1:** Management pathways.

	AR types			Aortic valve stenosis	Aortic root dilatation	STJ dilatation	SOV	Ascending aorta	Aortic arch	Surgical options
	1	2	3							
RHD			Y	Common	Not common	Not common	No	No	No	AVR, AVr
BAV		Y		85% of the cases	15% of the cases	Y	Y	Y	Y	AVR, AVr with or without reimplantation (Davids), remodeling
CTD	Y	Y		No	Common	Y	Y	Y	Y	AVR, AVr with or without reimplantation (Davids)
IE		Y		Not common	Possible	Possible	Possible	No	No	AVR
Degenerative			Y	Common	No	No	Possible	No	No	AVR
Congenital VSD		Y		No	No	No	No	No	No	AVr
Unicuspid valve			Y		Possible	Possible	Possible	Y	Y	AVR, AVr with or without reimplantation (Davids)

AR=aortic regurgitation; AVr=aortic valve repair; AVR=aortic valve replacement; BAV=bicuspid aortic valve; CTD=connective tissue disease; IE=infective endocarditis; RHD=rheumatic heart disease; SOV=sinus of Valsalva; STJ=sinotubular junction; VSD=ventricular septal defect; Y=yes

In vast majority of the developing nations of southeast Asia, South America, and Africa, rheumatic heart disease (RHD) is still the most common valvular pathology, and mitral valve involvement is the most common finding, followed by multivalvular lesions^[1]^. While in the developed Western world, RHD numbers are going down with increase in the degenerative valvular lesions (30-50%), and more often degenerative valves are stenotic lesions. Although the overall incidence of connective tissue disorder and congenital AV disease is stable (25-40%), the numbers of pure aortic regurgitation (AR) are limited, which could be a bottleneck in the learning curve for the surgeons^[14]^.

**Fig. 1:**
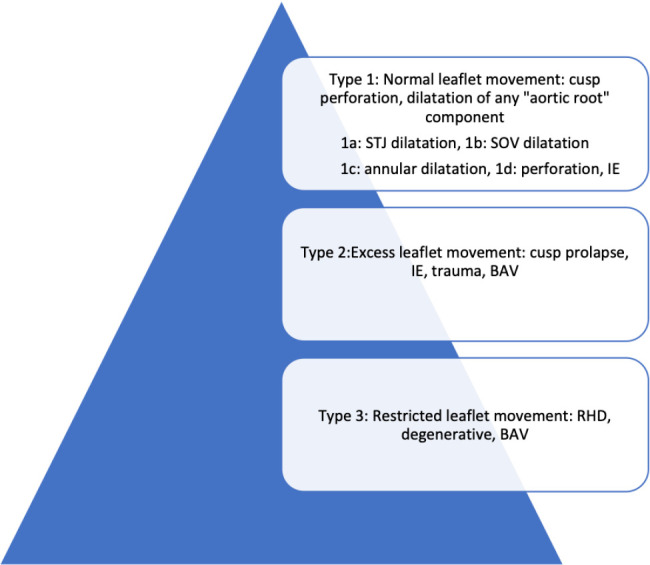
Classification of aortic regurgitation. BAV=bicuspid aortic valve; IE=infective endocarditis; RHD=rheumatic heart disease; SOV=sinus of Valsalva; STJ=sinotubular junction

Another very important aspect of the reliable AVr is to recognise intraoperative etiopathology comprehensively and to standardise findings for the proper reporting. AR has been classified in three groups to help the pathophysiology of the lesion and decide about type of repair (Figure 1)^[15]^. In a retrospective surgical study, pure valvular AR (type 1 lesion) was reported in 46% of the cases, while 54% of the patients had annular or ascending aortic dilatation (types 2 and 3 lesion)^[14]^. AVr outcomes are better in types 1 and 2 regurgitant lesions, while type 3 AR had poor outcomes because of poor tissue quality. Normal range of coaptation height is between 4-5 mm, while geometric height and effective height are 5 mm (BAV) and 9 mm, respectively^[15]^. All these finer technical points are part of a long learning curve, and if AVr is not performed regularly, then results might be suboptimum.

Anaesthetic experience in the intraoperative transoesophageal echocardiogram (TOE) is pivotal during AVr or aortic root procedures (remodeling or reimplantation) as it can guide towards better functional repair. De Waroux et al. have predicted “repairability” on the TOE and reported high reoperation rates (35% at the four-year follow-up) after AVr in the high-risk valve anatomy (type 3 lesions, type 2 lesion with severe prolapse, moderate calcification, significant leaflet restriction, valve coaptation < 4 mm, coaptation below the annular plane, and need for large pericardial patch extension)^[16]^.

#### Bicuspid Aortic Valve

**Fig. 2:**
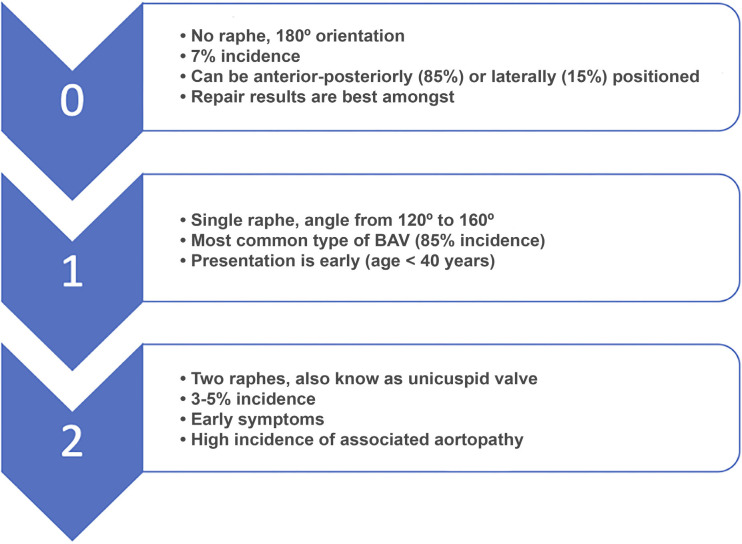
Bicuspid aortic valve classification. BAV=bicuspid aortic valve

Cusp morphology in the BAV can be heterogeneous, and Sievers et al. have used a practical classification to report BAV valves^[17]^. BAV prevalence is in the range of 1%-2% in the general population (Figure 2). Type 0 BAVs are less common, have two symmetric aortic sinuses (180 degrees) with two commissures, and do not contain a median raphe. The two most common patterns of cusp fusion in type 0 BAV disease are fusion of the left and right coronary cusps, which occurs in 70%-85% of cases, and fusion of the right and noncoronary cusps, which occurs in 15%-30% of patients with BAV. The mechanism of AR in this most commonly BAV with two cusps, two sinuses, and two commissures at approximately 180 degrees (type 0) is ideal for the AVr^[18]^. In these patients, usually the prolapse of one or both cusps and the dilatation of the FAA is the cause of AR (type 2 lesion).

The more prevalent type 1 BAVs have a median raphe on the conjoint cusp and an asymmetric distribution of the aortic sinuses, with a large aortic sinus accompanying a large nonconjoint cusp and two smaller cusps fused together with a median raphe. AR in type 1 valves can be due to a rigid and restrictive raphe associated with smaller fused cusps (type 3 lesion). Mostly, BAVs are associated with aortic stenosis (AS) (85%) and in only 15% of the cases, aortopathy (dilated STJ and ascending aorta) is seen. Incidence of pure valvular AR is not well reported, although often it is caused by the annular or ascending aortic dilatation^[19]^. Aortopathy phenotype patients are mostly male, and they present early with the symptoms. Freedom from late adverse ascending aortic events in the operated patients of AR associated with BAV has poor prognosis compared to the AS with BAV (93±3% *vs.* 78±9% at 15 years postoperatively, respectively)^[20]^.

#### Rheumatic Heart Disease

Mostly, type 1 and 2 lesions had better outcomes compared to RHD patients where major lesions are restriction and fibrosis^[13]^. Long-term outcomes of RHD valve repair are suboptimum and associated with high rate of reoperations^[21]^. Boodhwani et al.^[22]^ have reported a five-year survival of 95% and freedom from reoperation of 92% in type 1 and 2 lesions. However, freedom from reoperation was reduced in restricted (type 3) groups (88% *vs.* 94% in types 1 and 2).

In a series from India, results of the rheumatic AVr have been presented using various repair techniques, and the reported freedom from the moderate to severe AV disease was 82.5% at eight years and 52.5% at 13 years of follow-up.

Various repair techniques have been described for AVr (peeling, shaving, extension, decalcification, free edge plication, commissurotomy, and neo-cuspidization), and often they are reasonable in mild to moderately damaged valves, but results are poor in severely affected lesions^[23,24]^. Ozaki et al.^[25]^ have described a technique of leaflet neo-cuspidization using autologous pericardium and reported actuarial freedom from death, cumulative incidence of reoperation, and recurrence of the moderate AR as 85.9%, 4.2%, and 7.3%, respectively, at 54 months of follow-up. Ozaki technique can be an important method to create neo-leaflets in younger rheumatic patients where AVR might not be the best option. Various other groups have used this technique with few modifications and reported good mid-term to long-term outcomes^[26]^. Again, these highly specialized techniques will be hard to generalize for every cardiac unit and might lead to poor outcomes in the mid-term follow-up.

### Does Aortopathy Differs in Caucasian *vs*. Non-Caucasian Population?

Multiple meta-analysis has demonstrated potential long-term benefit of valve-sparing root replacement in cases of aortopathy in Caucasian patients, but on the contrary, long-term follow-up in Asian population are limited and occasional^[27,28]^.

The pattern of aortopathy in European and Asian population is significantly different as far as clinical features of the cardiovascular, ocular, and skeletal systems are concerned^[29]^. Asian Marfan populations have a higher prevalence of aortic root dilatation and mitral valve prolapse compared with Caucasian Marfan populations. Type 0 BAV was more frequently observed among Europeans compared with Asians (14.5% *vs*. 6.8%), whereas type 1 BAV with fusion raphe between the right and non-coronary cusps was more frequently observed in the Asian group compared with the European group (19.7% *vs*. 13.6%). In addition, the European group had higher prevalence of significant AR and diffusely dilated type of bicuspid aortopathy compared with the Asian group^[30]^.

Another North American study showed that aortic dilatation and severity of BAV were higher in the Caucasian communities compared to the African American patients^[31]^. Their findings raise some important issues regarding the role of genetic, ethnic, or other vascular modifiers which need to be studied in the future. Russo et al.^[32]^ have reported that patients who underwent surgery for type 1 BAV had more fibrosis, medio-necrosis, cystic medial necrosis, smooth muscle cell orientation, elastic fragmentation, and inflammation compared with other configurations in the follow-up.

It would be interesting to report differences in the aortopathy patterns from the different parts of the world and tailor-making surgical options based on those findings.

### How Common is Pure AR and How Many Cases Surgeons Need to Keep Competence?

The Global Burden of Disease study group had reported that isolated AS was the third, and pure AR was the fourth most common valve lesion in RHD cases^[33]^. Even in the Western world, referral for pure AR is not frequent in the regular cardiothoracic units, and it significantly reduces surgical team’s competency in giving standard expected outcomes.

Malas et al.^[34]^ have conducted a collaborative study with contribution from two busy AVr centres (one in Canada and another in Belgium) and reported about the learning curve to master the AVr technique. It takes approximately 40-60 cases to bring down complication rates, cardiopulmonary bypass time, aortic cross-clamping time, and to give standard reproducible outcomes. If surgeons are not getting regular referral, then they might take five to six years to perform the required numbers to overcome the steep learning curve.

### Standardization of AVr Reporting

Standardization of the reporting can be tedious because of various surgical strategies, different techniques, and variable reporting patterns of the results^[15]^. Comparing outcomes with proven technique like AVR can be challenging^[11]^. The Aortic Valve Insufficiency and ascending aorta Aneurysm InternATiOnal Registry (AVIATOR) was created in European centres for enrolling patients with this subset of pathology^[35]^. The reconstructive surgery includes isolated valve repair in 27%, partial root or tubular aorta replacement plus valve repair in 23%, and valve-sparing root replacements in 50% of the cases. Replacements include isolated valve replacement in 22%, tubular aorta plus AVR in 19%, and root plus valve replacement (Bentall) in 59% of the cases. Most of the expert European centres on AVr are reporting patients in the AVIATOR, while participation from American (9.5%) and Asian countries is low (0.5%), which again reflects on the discord in reporting.

### Follow-up and Reoperation

A multivariate analysis of the AVr failure in a series with 10-year follow-up using intraoperative TOE had identified a shorter coaptation length (< 4 mm), eccentric jet, coaptation occurring below the level of the aortic annulus, a larger aortic annulus, and residual AR (central jet > 3mm) at the end of surgery as high-risk factors of repair failure^[36]^. Type 3 repairs were especially at risk of AR recurrence because the leaflet tissue is either of not good quality (infective endocarditis, calcification, or rheumatic disease leading to the fibrosis) or insufficient to reconstruct the leaflet and require pericardial patching. Finally, another well reported pathological finding is the extension of native disease process in the residual repaired valve tissues and involves neo-cusp pericardial patch as well, eventually leading to AVr failure.

## CONCLUSION

AVr is a leap forward step in the treatment of AV and root pathology but requires a careful team-approach in the decision making and execution. The surgical community requires further long-term reports from various demographic regions of the world before generalizing the indications. Number of cases and associated learning curve is well-established with these techniques, which reinforces the need for regular referrals to specific centres and can expand surgical experience in handling the complication-associated failures and help in developing AVr as a subspecialty.
